# Qualitative and quantitative dermatoglyphic traits in patients with breast cancer: a prospective clinical study

**DOI:** 10.1186/1471-2407-7-44

**Published:** 2007-03-13

**Authors:** Chintamani, Rohan Khandelwal, Aliza Mittal, Sai Saijanani, Amita Tuteja, Anju Bansal, Dinesh Bhatnagar, Sunita Saxena

**Affiliations:** 1 Department of Surgery, Vardhman Mahavir Medical College, Safdarjang hospital, New Delhi, India; 2 Institute of Pathology, Indian Council Of Medical Research, Vardhman Mahavir Medical College, Safdarjang Hospital, New Delhi, India; 3 Vardhman Mahavir Medical College, Safdarjang Hospital, New Delhi, India

## Abstract

**Background:**

Breast cancer is one of the most extensively studied cancers and its genetic basis is well established. Dermatoglyphic traits are formed under genetic control early in development but may be affected by environmental factors during first trimester of pregnancy. They however do not change significantly thereafter, thus maintaining stability not greatly affected by age. These patterns may represent the genetic make up of an individual and therefore his/her predisposition to certain diseases. Patterns of dermatoglyphics have been studied in various congenital disorders like Down's syndrome and Kleinfelter syndrome. The prints can thus represent a non-invasive anatomical marker of breast cancer risk and thus facilitate early detection and treatment.

**Methods:**

The study was conducted on 60 histo-pathologically confirmed breast cancer patients and their digital dermatoglyphic patterns were studied to assess their association with the type and onset of breast cancer. Simultaneously 60 age-matched controls were also selected that had no self or familial history of a diagnosed breast cancer and the observations were recorded. The differences of qualitative (dermatoglyphic patterns) data were tested for their significance using the chi-square test, and for quantitative (ridge counts and pattern intensity index) data using the t- test.

**Results:**

It was observed that six or more whorls in the finger print pattern were statistically significant among the cancer patients as compared to controls. It was also seen that whorls in the right ring finger and right little finger were found increased among the cases as compared to controls. The differences between **mean pattern intensity index **of cases and controls were found to be statistically significant.

**Conclusion:**

The dermatoglyphic patterns may be utilized effectively to study the genetic basis of breast cancer and may also serve as a screening tool in the high-risk population. In a developing country like India it might prove to be an anatomical, non-invasive, inexpensive and effective tool for screening and studying the patterns in the high-risk population.

## Background

The genetic component in breast cancer is well established and various genes like (BRCA1 and BRCA2), p-53 etc. have been extensively studied and identified as genetic links [1-3]. Evidence is available suggesting that a family history of breast cancer might be associated with a specific fingerprint pattern [4-8]. Finger print determination is genetic but has been reported to be affected by the environmental factors in the first trimester of pregnancy. After birth the patterns remain more or less constant and hence may serve to study the genetic patterns in any individual [9]. The fingerprints could thus be used for screening or to guide future research in this direction and one day the screening of breast cancers could well be at our fingertips!. The finger and palmar print patterns have already been studied with respect to various genetic diseases like the Down's syndrome and Klinefelter syndrome [4,5]. The prints can thus represent a noninvasive anatomical marker of breast cancer risk [4-6]. An effort in this regard has been made to devise a screening program for breast cancer using fingerprints or dermatoglyphic study in order to select the high-risk group for surveillance.

## Methods

The study was conducted on 60 histo-pathologically confirmed breast cancer patients after taking the informed consent and permission from the institutional review board of Safdarjang hospital Ministry of health India. The patients were asked to fill a Performa and their digital prints recorded. The fingerprints were taken on a glossy paper by rolling finger technique using ink, digitally photographed and analyzed using Adobe Photoshop software for palmar ridge patterns and ridge counts. Simultaneously 60, age matched controls were also selected that had no self or familial history of diagnosed breast cancer and their observations were also recorded. The difference of qualitative (dermatoglyphic pattern) data was tested for its significance using the chi-square test, and for quantitative (Ridge counts and Pattern intensity index) data using the t-test.

## Results

The two most common patterns for a digit were recorded for keeping the numbers optimum and only for more clarity in interpretation of the data.

The mean ridge count in the right hand of cases was 12.4 whereas it was 18.4 in the controls. The standard deviation of the ridge count in the right hand of cases was 2.33 and the standard deviation in the right hand of controls was 4.58. When t-test was applied using SPSS-Version 11, the difference in the mean ridge count of cases and controls was significant in the right hand. (p < 0.05) (Table. 1).

The mean ridge count in the left hand of cases was 12.4 whereas it was 19.6 in the controls. The standard deviation of the ridge count in the left hand of cases was 1.62 and the standard deviation in the left hand of controls was 4.67. When t-test was applied using SPSS-Version 11, the difference in the mean ridge count of cases and controls was significant in the left hand (p < 0.05) (Table. 2).

The mean pattern intensity index in cases was 12.91 compared to 11.33 for the controls. The difference in the pattern intensity index for these cases was found to be statistically significant with p < 0.03 and t = 2.10 (CI – 1.59) (Table. 3).

The following observations were made with respect to qualitative parameters (dermatoglyphic patterns)(Table. 4)

a) 6 or more whorls were found to be significantly higher in cases as compared to controls (X^2 ^- 5.71 df-1 p < 0.02)

b) Whorls were commonly observed in right ring finger in cases as compared to controls (X^2 ^- 5.67 df-1 p < 0.02).

c) Whorls were more commonly observed in right little finger in cases as compared to controls (X^2 ^- 7.67 p < 0.01).

Out of 60 cases, 57 had infiltrating breast carcinoma, 2 had inflammatory carcinoma and 1 had breast sarcoma. Of the 2 cases with inflammatory carcinoma, 1 patient had 6 digital whorls whereas the patient with breast sarcoma did not have 6 or more whorls. Of the 57 cases, 23 had 6 or more digital whorls (40.53%) (Table. 5)

## Discussion

More has been written about the epidemiology of breast cancer than possibly any other form of cancer affecting mankind [7]. However, in the face of this intense interest, only a paucity of attention has been given to the role of genetics in its etiology [7-11]. Familial clustering of breast cancer was first recorded in the Roman medical literature at around 100AD [8].

**Figure 1:**
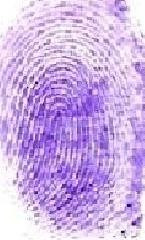
The loop pattern in control.

**Figure 2:**
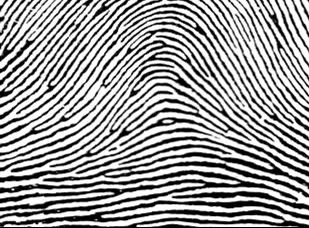
The whorl pattern in a breast cancer patient.

**Figure 3:**
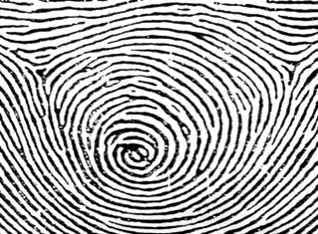
The arch pattern in a control.

In some studies a pattern of six or more digital whorls was recorded more frequently in women with breast cancer than in those without the disease [9,10]. The presence of six or more whorls was found to be significant as noted by 32.4% of breast cancer patients possessing this number of whorls as compared to 3.1% controls. Also of note is that 95% of subjects with six or more whorls either had cancer or were at high-risk. Similar results were obtained in the present study. Loops, arches and whorls are the common patterns observed in individuals (figures 1, 2, 3).

Presence of Whorl pattern in the present study is also important for a different reason. It is seen that the whorl pattern frequency showed maximal changes as compared to other patterns i.e. 4 % increase in the right digits in cancer patients as compared to controls.

Moreover the ridge count was also considered for correlation, as it is more objective and easier to assess. In the present study it was observed that the ridge count was significantly lower in cases as compared to controls. It was observed that the mean ridge count on the right hand and left hand was 10.4 and 12.4 respectively, while in the controls the ridge count in the ridge and left hand was observed to be 18.4 and 19.6 respectively. It was found to be statistically significant (p < 0.05).

However in another study a totally different observation was noted in the form of six or more whorls being protective of breast cancer, contrary to that observed in other studies and the present study. Although this study was also conducted in the Indian setting, it have something to do with the sample size and geographic distances or due to the different ethnic group being included in the study (Goans and south Indian Christians) contrary to the present study where all patients were North Indian Hindus [10,11].

This has also been suggested by Gilligan et al (1985) where a significant correlation between dermatoglyphic and geographic distances was found confirming the biological validity of the social and ethnic criteria [4,11]. This gives us more reasons to work on these patterns extensively to come to a conclusive statement about our population for application in the field and we indeed need "a local solution to a local problem".

Although limited conclusions could be drawn based on this preliminary study, digital dermatoglyphics may have a future role in identifying women either with or at increased risk for breast cancer so that either risk reduction measures or earlier therapy may be instituted and we also have some evidence from this preliminary study to suggest that a family history of breast cancer might be associated with a specific fingerprint patterns which may be used as a potential non-invasive anatomical tool to be used for screening for breast cancer and for guiding future research.

This relatively non-invasive technique can reasonably be used in selective non-symptomatic women (those with positive family history) as a part of definite risk assessment strategy with an ability to detect the earliest changes associated with tumorogenesis many years before the appearance of measurable tumor and this may allow the introduction of more effective chemopreventive strategies and early diagnosis and treatment in patients with breast cancer.

## Conclusion

In the present study it was observed that the ridge count is significantly lower in cases as compared to controls and the whorl pattern frequency showed maximal changes as compared to other patterns i.e. 4 % increase in the right digits in cancer patients as compared to controls. Though a high-risk population is epidemiologically identified, these studies might help us to identify the possibility of breast cancer to take preventive measures concerning the environmental factors and particularly the hormonal factors. Women at a high risk of breast cancer would have many options available to them including prophylactic mastectomy, watchful waiting, chemoprevention if the risk could be assessed accurately.

The study is ongoing and the pattern seems to be appearing wherein a definite approach in the form of "dermatoglyphics" might play a significant role in the near future not only for the purpose of screening but also for studying the behavior of breast cancer.

## Competing interests

The author(s) declare that they have no competing interests.

## Authors' contributions

CM, the principal author designed and conceptualized the study, RK, AM, AT, S, DB assisted in the data collection and statistical analysis, AB and SS performed the histopathological examinations and assisted in the designing of protocols. All authors read and approved the manuscript.

## Pre-publication history

The pre-publication history for this paper can be accessed here:



## Figures and Tables

**Table 1:** Mean ridge count in the right hand of breast cancer patients & controls

Digit	Ridge count -cases	Ridge count -controls
Thumb	16	18
Index	11	25
Middle	13	14
Ring	13	22
Little	9	13

Mean ridge count in cases = 12.4, controls = 18.4

Ridge count S.D. cases = 2.33, controls = 4.58

The difference was found to be statistically significant (p < 0.05) *t = 2.33 (CI -6.00)*

**Table 2:** Mean ridge count in the left hand of breast cancer patients & controls

Digit	Ridge count -cases	Ridge count -controls
Thumb	13	18
Index	8	25
Middle	10	14
Ring	10	22
Little	11	13

Mean ridge count cases = 12.4, controls = 19.6

Ridge count S.D. cases = 1.62, controls = 4.67

The difference was found to be statistically significant (p < 0.05) *t *= *3.28 (CI -8.00)*

The **pattern intensity index **of breast cancer patients as well as the controls was studied & the following observations were made.

**Table 3:** 

	Cases	Controls
Mean	12.91	11.33

t = 2.10 (CI – 1.59) p < 0.03

**Table 4:** Frequency of each digital pattern.

	Right digits in breast cancer patients	Left digits in breast cancer patients	Right digits in controls	Left digits in controls
Loops	75.0	75.4	48.2	19
Whorl	4.0	3.0	-	6
Double Loop	8.0	-	16.0	10.4
Tented Arch	6.0	14.8	20.0	29

**Table 5:** Division of cases on the basis of histological types:

Histological types (n = 60)	Number of cases	Number of cases with 6 or more whorls
Infiltrating carcinoma (IDC)	57 (95%)	23
Inflammatory carcinoma	2 (3.33%)	1
Breast sarcoma	1 (1.67%)	0
